# 

**Published:** 1888-12

**Authors:** 


					﻿vol viii.
DECEMBER, 1888.
NO. 12S
juhe
HOMEOPATHIC PHYSICIAN,
A Monthly Journal of Homoeopathic Materia Medica
and Clinical Medicine.
“He is a freeman whom truth makes free, and all are slaves beside.”
“Seek the Truth: come whence it may, cost what it will.”
EDITED AND PUBLISHED BY
Edmund j. lee, m. d.,
AND
WALTER Nf. JAMES, M. 13.
PHILADELPHIA:
No. 1123 8PRUCE STREET.
T. O 2ST D O 3ST :
ALFRED HEATH & CO., Sole Agents for Great Britain,
114 Ebury Street, Eaton Square.
DS* Yearly Subscription, $2.50. invariably in advance. Single numbers, 25 cts.
Entered at the Philadelphia Poet-Office as Second-Class Mall Matter.
				

